# Comparison of Clinical Outcomes Among Patients With Atrial Fibrillation or Atrial Flutter Stratified by CHA_2_DS_2_-VASc Score

**DOI:** 10.1001/jamanetworkopen.2018.0941

**Published:** 2018-08-03

**Authors:** Yu-Sheng Lin, Yung-Lung Chen, Tien-Hsing Chen, Ming-Shyan Lin, Chi-Hung Liu, Teng-Yao Yang, Chang-Ming Chung, Mien-Cheng Chen

**Affiliations:** 1 Division of Cardiology, Department of Internal Medicine, Chang Gung Memorial Hospital, Chiayi, Taiwan; 2 Chang Gung University College of Medicine, Taoyuan, Taiwan; 3 Graduate Institute of Clinical Medical Sciences, College of Medicine, Chang Gung University, Taoyuan, Taiwan; 4 Division of Cardiology, Department of Internal Medicine, Kaohsiung Chang Gung Memorial Hospital, Chang Gung University College of Medicine, Taiwan; 5 Division of Cardiology, Department of Medicine, Chang Gung Memorial Hospital, Keelung, Taiwan; 6 Stroke Center and Department of Neurology, Chang Gung Memorial Hospital, Linkou Medical Center, Chang Gung University College of Medicine, Taoyuan, Taiwan

## Abstract

**Importance:**

Current guidelines support treating atrial fibrillation (AF) and atrial flutter (AFL) as equivalent risk factors for ischemic stroke stratified by CHA_2_DS_2_-VASc scores, recommending anticoagulation therapy for patients with a CHA_2_DS_2_-VASc score of 2 or higher, but some studies found differences in clinical outcomes.

**Objective:**

To investigate differences in clinical outcomes among AF, AFL, and matched control cohorts.

**Design, Setting, and Participants:**

This nationwide cohort study analyzed data from the Taiwan National Health Insurance Research Database from January 1, 2001, through December 31, 2012. Follow-up and data analysis ended December 31, 2012. A total of 219 416 age- and sex-matched individuals participated in the study. Clinical outcomes were compared after stratification by CHA_2_DS_2_-VASc score (possible score range, 0-9; higher scores indicate greater risk of ischemic stroke).

**Main Outcomes and Measures:**

Ischemic stroke, heart failure hospitalization, and all-cause mortality among the AF, AFL, and matched control cohorts were analyzed using Cox proportional hazards regression.

**Results:**

This study comprised 188 811 patients in the AF cohort (mean [SD] age, 73.8 [13.4] years; 104 703 [55.5%] male), 6121 patients in the AFL cohort (mean [SD] age, 67.7 [15.8] years; 3735 [61.0%] male), and 24 484 patients in the matched control cohort (mean [SD] age, 67.3 [15.6] years; 14 940 [61.0%] male). The patients with AF were older, were more predominantly female, and had higher CHA_2_DS_2_-VASc scores than the patients with AFL and the control participants. After stratification by CHA_2_DS_2_-VASc score, the incidence densities (IDs; events per 100 person-years) of ischemic stroke (AF cohort: ID, 3.08; 95% CI, 3.03-3.13; AFL cohort: ID, 1.45; 95% CI, 1.28-1.62; controls: ID, 0.97; 95% CI, 0.92-1.03), heart failure hospitalization (AF cohort: ID, 3.39; 95% CI, 3.34-3.44; AFL cohort: ID, 1.57; 95% CI, 1.39-1.74; controls: ID, 0.32; 95% CI, 0.29-0.35), and all-cause mortality (AF cohort: ID, 17.8; 95% CI, 17.7-17.9; AFL cohort: ID, 13.9; 95% CI, 13.4-14.4; controls: ID, 4.2; 95% CI, 4.1-4.4) were significantly higher in the AF cohort than in the matched control cohort. For the AFL cohort vs the matched control cohort, the incidences of heart failure hospitalization and all-cause mortality were significantly higher across all levels, but the incidence of ischemic stroke was only significantly higher at CHA_2_DS_2_-VASc scores of 5 to 9. For the AF cohort vs the AFL cohort, the incidences of ischemic stroke and heart failure hospitalization were significantly higher at a CHA_2_DS_2_-VASc score of 1 or higher, but the incidence of all-cause mortality was significantly higher only at CHA_2_DS_2_-VASc scores of 1 to 3.

**Conclusions and Relevance:**

This study found different clinical outcomes between patients with AFL and AF and those without AF and AFL. The current recommended level of the CHA_2_DS_2_-VASc score in preventing ischemic stroke in patients with AFL should be reevaluated.

## Key Points

**Question:**

Do clinical outcomes differ between atrial fibrillation and atrial flutter by CHA_2_DS_2_-VASc scores?

**Findings:**

This nationwide cohort study of 219 416 age- and sex-matched individuals in Taiwan found that the incidence of ischemic stroke among patients with atrial flutter was significantly lower than that among patients with atrial fibrillation at a CHA_2_DS_2_-VASc score less than 5, whereas the incidences of heart failure hospitalization and all-cause mortality were different across different CHA_2_DS_2_-VASc scores.

**Meaning:**

The current recommended level of CHA_2_DS_2_-VASc score (≥2) used to prevent ischemic stroke in patients with atrial flutter should be reevaluated and prospectively studied.

## Teaser

This cohort study uses Taiwan National Health Insurance database data to evaluate the incidence of ischemic stroke, hospitalization for heart failure, and all-cause mortality among patients with atrial fibrillation and atrial flutter stratified by CHA_2_DS_2_-VASc score.

## Introduction

Atrial flutter (AFL) and atrial fibrillation (AF) are often grouped together in terms of risk stratification and in epidemiologic studies.^1,2^ The incidence of AFL is approximately one-sixteenth that of AF.^3^ The incidence of AFL was reported to be 88 per 100 000 people, and the incidence of solitary AFL was reported to be 37 per 100 000 person-years in the general population during the 4-year observational study of the Marshfield Epidemiologic Study Area.^4^ Atrial flutter is similar to AF in that its incidence increases with age^4,5^ and it contributes to heart failure, stroke, and all-cause mortality.^3,6^ Therefore, the pharmacologic management of AFL is usually considered to be the same as for AF, especially for preventing thromboembolic events.^7^ Although AF and AFL share many common risk factors for occurrence,^4,5,8,9^ differences in clinical outcomes have been reported. For example, one study^10^ reported a higher incidence of mortality among patients with AF than among those with AFL during a 7-year observation period, and another study^11^ reported a lower incidence of stroke among patients with solitary AFL compared with those with AF. Although the Framingham Heart Study^3^ found that AF and AFL were associated with equal outcome of stroke, findings are weakened by the small study population.

The CHA_2_DS_2_-VASc scoring system was developed in 2010^12^ and is calculated according to a point system in which 2 points are assigned for a history of stroke or transient ischemic attack (S_2_) or age (A_2_) older than 75 years and 1 point each is assigned for an age (A) of 65 to 74 years or a history of congestive heart failure (C), hypertension (H), diabetes (D), vascular disease (V) (myocardial infarction and peripheral artery disease), and female sex (sex category [Sc]). CHA_2_DS_2_-VASc is currently the standard scoring system for risk stratification to predict thromboembolic events in patients with AF and AFL and is a means of stratifying the risk of hospitalization for heart failure and mortality in these patients.^13,14^ On the basis of the distinct underlying electrophysiologic mechanisms and myocardial substrates of AFL and AF,^15,16^ the incidence of ischemic stroke, hospitalization for heart failure, and all-cause mortality would be expected to be different across different levels of CHA_2_DS_2_-VASc score. Therefore, we conducted this study to evaluate the incidence of ischemic stroke, hospitalization for heart failure, and all-cause mortality among patients with AF and AFL stratified by levels of CHA_2_DS_2_-VASc score in a large, population-based national database.

## Methods

### Data Source

This retrospective nationwide cohort study analyzed data from the Taiwan National Health Insurance Research Database (NHIRD) that were released by the Taiwan National Health Research Institutes from January 1, 1997, to December 31, 2012. The NHIRD contains health care information of more than 23 million Taiwan residents who are enrolled in the mandatory National Health Insurance program^17,18^; the database includes registration and demographic data, drug prescriptions, interventions and examinations, complete outpatient clinic visits, hospitalizations, vital status, and diseases, which are registered using *International Classification of Diseases, Ninth Revision, Clinical Modification* (*ICD-9-CM*) codes. In the NHIRD, the identification numbers of the patients are encrypted to protect their privacy, and the encryption procedure is consistent so that linking claims belonging to the same enrollee is feasible and can be followed longitudinally. This study was approved by the institutional review board of Chang Gung Memorial Hospital, and the need for written informed consent was waived by the ethics committee. This study followed the Strengthening the Reporting of Observational Studies in Epidemiology (STROBE) reporting guideline.

### AF, AFL, and Matched Control Cohorts

**Figure 1.**
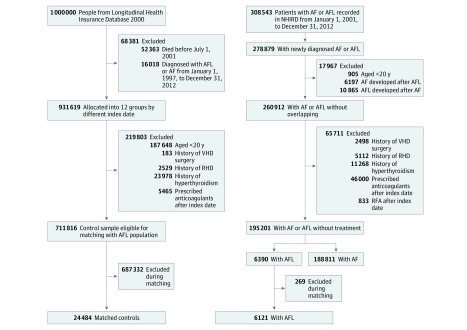
Flowchart of the Study Design AF indicates atrial fibrillation; AFL, atrial flutter; NHIRD, National Health Insurance Research Database; RFA, radiofrequency ablation; RHD, rheumatic heart disease; and VHD, valvular heart disease.

The diagnoses of AF and AFL were confirmed by more than 2 outpatient diagnoses or 1 inpatient diagnosis in the NHIRD, as validated in previous studies.^19,20^ The index date was defined as the date when AF or AFL was first diagnosed. After excluding 246 patients with missing information, 308 543 patients with newly diagnosed AF or AFL from January 1, 2001, to December 31, 2012, were identified, and those 20 years or older were enrolled in this study. After excluding the patients with AF with a concomitant diagnosis of AFL and those with AFL with a concomitant diagnosis of AF during the observation period, we identified 260 912 adult patients with a diagnosis of solitary AFL or solitary AF (Figure 1). We also excluded patients with rheumatic heart diseases, those who underwent surgery for valvular heart diseases, and those with reversible causes of AF and AFL, such as hyperthyroidism and coexisting sepsis or heart surgery when AF or AFL was diagnosed during the same hospitalization. Patients who received therapy that may have had an influence on the study outcomes during the observation period, such as radiofrequency catheter ablation for AF or AFL and anticoagulation therapy, were also excluded. The underuse of oral anticoagulants for patients with nonvalvular AF in Asia (prevalence of approximately 20%-30%), including in Taiwan,^21,22^ provided the opportunity to evaluate patients with AF and AFL who did not receive adequate stroke prevention therapy, thereby minimizing selection bias. Therefore, the patients prescribed anticoagulants after the index date were also excluded. A final total of 188 811 patients were enrolled in the solitary nonvalvular AF cohort, with 6390 in the solitary nonvalvular AFL cohort (Figure 1).

The control participants were defined as those without a diagnosis of AF or AFL between January 1, 1997, and December 31, 2012, and were selected from the 2000 Longitudinal Health Insurance Database, which provides longitudinally linked anonymized data of 1 000 000 enrollees (nearly 5% of the population in Taiwan) randomly sampled from the 2000 Registry for Beneficiaries of the NHIRD. The Longitudinal Health Insurance Database has been validated as a representative sample of the Taiwanese population in terms of age, sex, and mean payroll bracket.^23^ The control participants were randomly divided into 12 subgroups according to different index dates, with the middle year set as the index date (eg, July 1, 2001, July 1, 2002, and so on until July 1, 2012) in each subgroup. The exclusion criteria for the control group were age younger than 20 years, rheumatic heart diseases or hyperthyroidism, surgery for valvular heart diseases during the observation period, and anticoagulation therapy after the index date. A final total of 711 816 control participants were eligible for age and sex matching with the 6390 patients with AFL because the AFL population was usually older than those without AFL or AF, and 24 484 control participants who were exactly matched using a 4:1 ratio to the AFL cohort by age, sex, and index year composed the matched control cohort (Figure 1).

### Outcome Assessment According to CHA_2_DS_2_-VASc Score

The study participants were followed up and data analyzed from the index date until a defined clinical outcome occurred or until December 31, 2012. Three clinical outcomes were evaluated: ischemic stroke, hospitalization for heart failure, and all-cause mortality. Hospitalization for heart failure and ischemic stroke were defined according to the principal diagnosis at admission. All-cause mortality was defined as withdrawal from the National Health Insurance program.^24^ The clinical outcomes in the 3 groups (AF, AFL, and control cohorts) were compared across different CHA_2_DS_2_-VASc levels. The lowest score is 0 and the highest score is 9 in the CHA_2_DS_2_-VASc scoring system, with higher scores indicating greater risk of ischemic stroke. This scoring system was also used in an Asian population,^25,26^ and the distribution of incidence of ischemic stroke was comparable with that in other studies.^27,28^ Each patient was categorized into a CHA_2_DS_2_-VASc level (0, 1, 2, 3, 4, and 5-9) because the age and sex components of CHA_2_DS_2_-VASc were already matched between the control and AFL cohorts.

### Ascertainment of AF, AFL, Comorbidities, and Outcomes

Atrial fibrillation (*ICD-9-CM* code 427.31), AFL (*ICD-9-CM* code 427.32), and all comorbidities were defined according to the diagnoses made during hospitalization or in at least 2 consecutive clinic visits (eTable 1 in the Supplement). The high accuracy of the diagnosis of AF based on *ICD-9-CM* coding in the NHIRD has been confirmed in a previous study,^29^ and a positive predictive value for the diagnosis of AFL of 97.5% was reported previously.^30^ The other comorbidities as reported in the literature^31,32^ were also validated, of which hypertension, diabetes, and dyslipidemia were diagnosed according to *ICD-9-CM* code and the use of related medications to increase the diagnostic accuracy (eTable 2 in the Supplement). In terms of study outcomes, we previously validated the principal diagnoses of heart failure and ischemic stroke at the index admission in patients with AF and AFL,^30^ and the positive predictive values were 94.2% for ischemic stroke and 97.6% for hospitalization for heart failure.

### Statistical Analysis

The patients’ clinical characteristics (ie, age, sex, baseline comorbidities, and medications) were compared among the 3 groups (AF vs AFL vs matched control cohorts) using 1-way analysis of variance for continuous variables or the χ^2^ test for categorical variables. For pairwise comparisons between any 2 study groups, we used Bonferroni adjustment. The risks of clinical outcomes (ischemic stroke, hospitalization for heart failure, and all-cause mortality) were expressed as incidence densities (IDs), defined as the number of events per 100 person-years. The approximate 95% CIs of the IDs were then calculated based on Poisson distribution. Comparisons of the incidence of clinical outcomes between any 2 cohorts were made using Cox proportional hazards regression analysis in pooled CHA_2_DS_2_-VASc score levels (score range, 0-9) or at a stratified CHA_2_DS_2_-VASc level (0, 1, 2, 3, 4, and 5-9). In addition, the proportion of each CHA_2_DS_2_-VASc component was also compared among the 3 cohorts using the χ^2^ test or 1-way analysis of variance with Bonferroni corrections. For the primary analysis (Cox proportional hazards regression), we conducted a sensitivity analysis in which patients who received any antiarrhythmic drugs after the index date were excluded. Because the patients with AF were older by a mean of 6 years than the patients with AFL, a falsification analysis was conducted to detect residual confounding.^33^ We chose hepatocellular carcinoma as the falsification end point because of its high incidence in Taiwan and no evidence of difference between AF and AFL cohorts. Levels of statistical significance were set as 2-sided *P* < .05, and no adjustment of multiple testing (multiplicity) was made in this study. SAS software for Windows, version 9.4 (SAS Institute Inc) was used for all statistical analyses. Participants with missing demographic information (ie, sex and date of birth) were excluded from the analysis (<0.1%). No participants were unavailable for follow-up.

## Results

### Baseline Characteristics of the AF, AFL, and Matched Control Cohorts

A total of 219 416 age- and sex-matched individuals participated in the study. There were 188 811 patients in the AF cohort (mean [SD] age, 73.8 [13.4] years; 104 703 [55.5%] male), 6121 patients in the AFL cohort (mean [SD] age, 67.7 [15.8] years; 3735 [61.0%] male), and 24 484 patients in the matched control cohort (mean [SD] age, 67.3 [15.6] years; 14 940 [61.0%] male). The AF cohort was older, was more predoninantly female, and had a significantly higher prevalence of comorbidities, including history of stroke, compared with the other 2 cohorts (Table). The AF and AFL cohorts had significantly higher prevalence rates of all comorbidities (Table) and a history of stroke and/or thromboembolic events and myocardial infarction compared with the control cohort, and this observation was consistent with the difference in the use of related medications (eTable 3 in the Supplement). In terms of CHA_2_DS_2_-VASc score, the AF cohort had the highest mean CHA_2_DS_2_-VASc score, whereas the control cohort had the lowest score (Table).

**Table.** Baseline Characteristics of the AF, AFL, and Matched Control Cohorts^a^

Characteristic	Matched Control Cohort (n = 24 484)	AFL Cohort (n = 6121)	AF Cohort (n = 188 811)
Age, mean (SD), y	67.3 (15.6)	67.7 (15.8)	73.8 (13.4)^b^^,^^c^
Age group, y			
<65	8764 (35.8)	2191 (35.8)	41 199 (21.8)^b^^,^^c^
65-74	6008 (24.5)	1502 (24.5)	43 340 (23.0)^b^^,^^c^
≥75	9712 (39.7)	2428 (39.7)	104 272 (55.2)^b^^,^^c^
Sex			
Male	14 940 (61.0)	3735 (61.0)	104 703 (55.5)^b^^,^^c^
Female	9544 (39.0)	2386 (39.0)	84 108 (44.5)^b^^,^^c^
Comorbidities			
Hypertension	8486 (34.7)	3248 (53.1)^b^	108 094 (57.2)^b^^,^^c^
Diabetes	3173 (13.0)	1201 (19.6)^b^	36 086 (19.1)^b^
Ischemic heart disease	2766 (11.3)	2115 (34.6)^b^	70 381 (37.3)^b^^,^^c^
Dyslipidemia	2018 (8.2)	775 (12.7)^b^	20 408 (10.8)^b^^,^^c^
Chronic obstructive pulmonary disease	1835 (7.5)	1208 (19.7)^b^	43 897 (23.2)^b^^,^^c^
Gout	1512 (6.2)	594 (9.7)^b^	18 739 (9.9)^b^
Abnormal liver function	1621 (6.6)	749 (12.2)^b^	21 119 (11.2)^b^^,^^c^
Malignant tumor	1209 (4.9)	543 (8.9)^b^	14 903 (7.9)^b^^,^^c^
Heart failure	579 (2.4)	798 (13.0)^b^	27 716 (14.7)^b^^,^^c^
Peripheral arterial disease	347 (1.4)	163 (2.7)^b^	5128 (2.7)^b^
Renal status			
Nonchronic kidney disease	23 387 (95.5)	5113 (83.5)^b^	159 348 (84.4)^b^
Chronic kidney disease without dialysis	933 (3.8)	762 (12.4)^b^	23 422 (12.4)^b^
Chronic kidney disease with dialysis	164 (0.7)	246 (4.0)^b^	6041 (3.2)^b^^,^^c^
History of disease			
Stroke or systemic thromboembolism	1995 (8.1)	968 (15.8)^b^	35 845 (19.0)^b^^,^^c^
Stroke	1881 (7.7)	868 (14.2)^b^	32 823 (17.4)^b^^,^^c^
Myocardial infarction	360 (1.5)	377 (6.2)^b^	9165 (4.9)^a^^,^^b^
CHA_2_DS_2_-VASc score, mean (SD)	2.2 (1.7)	3.0 (1.9)^b^	3.5 (1.9)^b^^,^^c^
CHA_2_DS_2_-VASc group			
0	4047 (16.5)	538 (8.8)	10 738 (5.7)
1	5458 (22.3)	1040 (17.0)	18 348 (9.7)
2	5191 (21.2)	1095 (17.9)	29 782 (15.8)
3	4490 (18.3)	1132 (18.5)	39 058 (20.7)
4	2847 (11.6)	1024 (16.7)	37 423 (19.8)
5-9	2451 (1.0)	1292 (21.1)	53 462 (28.3)

Abbreviations: AF, atrial fibrillation; AFL, atrial flutter.

^a^ Data are presented as number (percentage) of participants unless otherwise indicated.

^b^ Significant post hoc comparison vs the control group.

^c^ Significant post hoc comparison vs the AFL group.

### Ischemic Stroke

**Figure 2.**
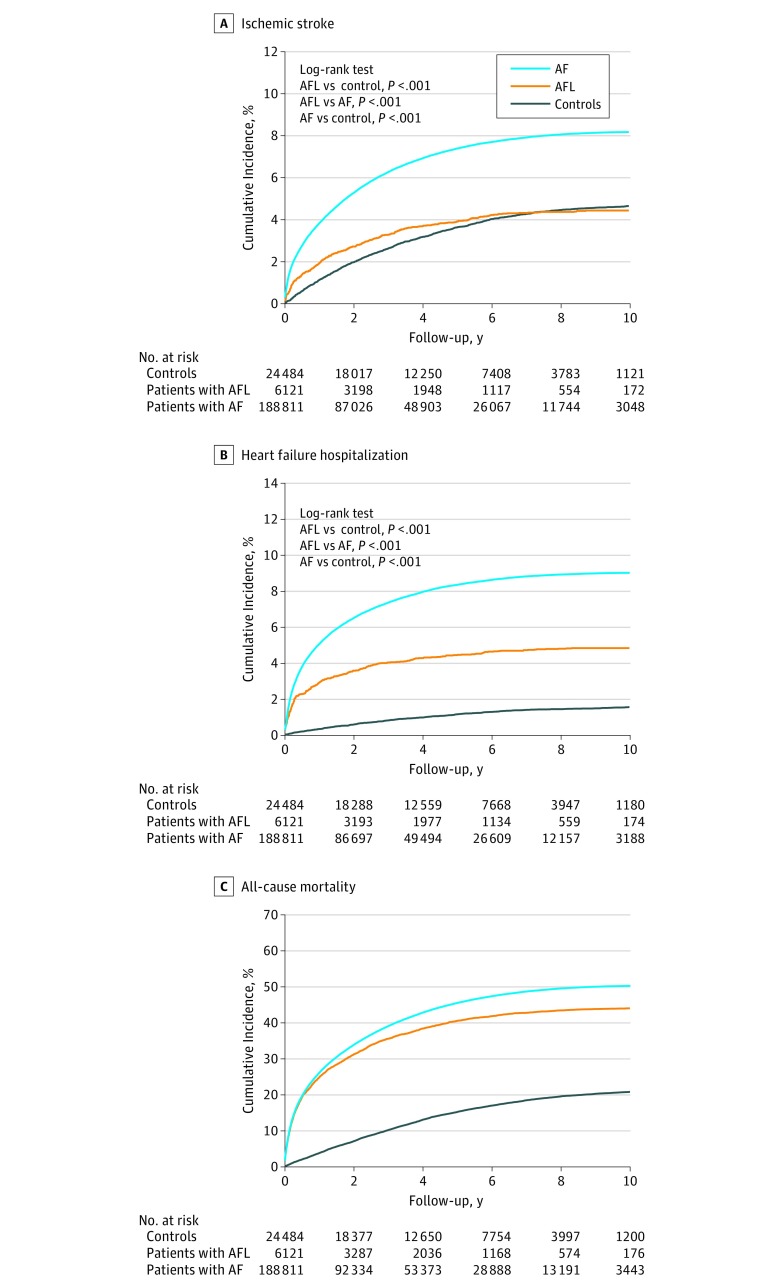
Kaplan-Meier Curves for Ischemic Stroke, Hospitalization for Heart Failure, and All-Cause Mortality in the Atrial Fibrillation (AF), Atrial Flutter (AFL), and Matched Control Groups in Real-world Conditions

**Figure 3.**
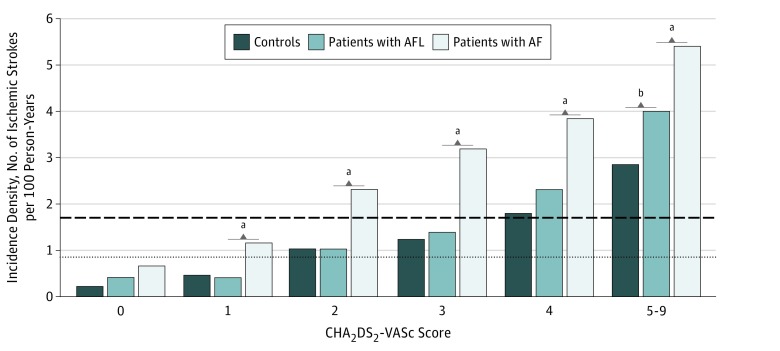
Incidence Density of Ischemic Stroke Among the Atrial Fibrillation (AF), Atrial Flutter (AFL), and Matched Control Cohorts With Different CHA_2_DS_2_-VASc Scores Dashed line indicates the cutoff value of the annual incidence (1.7%) needed to be prescribed anticoagulation drugs; dotted line, the cutoff value of the annual incidence (0.9%) needed to be prescribed non–vitamin K oral anticoagulants according to the study by Eckman et al.^34^ ^a^*P* < .001 for AF vs AFL cohorts. ^b^*P* < .001 for AFL vs matched control cohorts.

**Figure 4.**
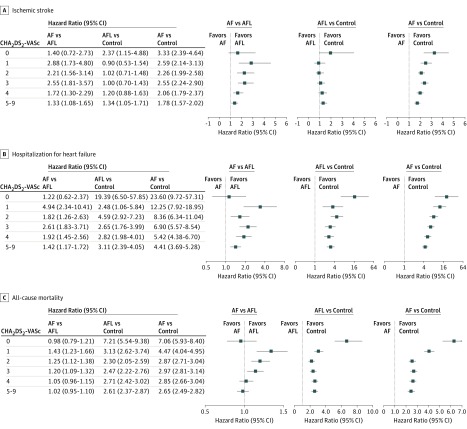
Comparisons of Clinical Outcomes Among the Atrial Fibrillation (AF), Atrial Flutter (AFL), and Control Cohorts Across Different CHA_2_DS_2_-VASc Scores Error bars indicate 95% CIs.

With a mean (SD) follow-up of 3.1 (2.9) years, the IDs of ischemic stroke were 3.08 (95% CI, 3.03-3.13) in the AF cohort, 1.45 (95% CI, 1.28-1.62) in the AFL cohort, and 0.97 (95% CI, 0.92-1.03) in the control cohorts, and there were significant differences among them (Figure 2A). When stratifying the groups by CHA_2_DS_2_-VASc score, the IDs of ischemic stroke increased with the level of CHA_2_DS_2_-VASc score in all 3 cohorts (Figure 3).^34^ In addition, the ID of ischemic stroke at a CHA_2_DS_2_-VASc score of 1 in the AF cohort (ID, 1.14; 95% CI, 1.06-1.22) was similar to that at a CHA_2_DS_2_-VASc score of 2 in the AFL cohort (ID, 1.02; 95% CI, 0.69-1.34) (Figure 3 and Figure 4A). Moreover, the ID of ischemic stroke at a CHA_2_DS_2_-VASc score of 2 in the AF cohort (ID, 2.30; 95% CI, 2.20-2.40) was similar to that at a CHA_2_DS_2_-VASc score of 4 in the AFL cohort (ID, 2.30; 95% CI, 1.72-2.88) (Figure 3 and Figure 4A). The ID of ischemic stroke in the AF cohort (ID, 3.99; 95% CI, 3.23-4.76) was significantly higher across all levels of CHA_2_DS_2_-VASc score compared with the control cohort, whereas the ID of ischemic stroke in the AFL cohort (ID, 2.83; 95% CI, 2.48-3.18) was only significantly higher at CHA_2_DS_2_-VASc scores of 5 to 9 (hazard ratio, 1.29; 95% CI, 1.02-1.62; *P* = .03) compared with the control cohort (Figure 4A). The ID of ischemic stroke was significantly higher in the AF cohort than in the AFL cohort across nearly all levels except at a CHA_2_DS_2_-VASc score of 0 (Figure 4A). The detailed information of comparison of ischemic stroke is provided in eTable 4 in the Supplement.

### Hospitalization for Heart Failure

The IDs for hospitalization for heart failure were 3.39 (95% CI, 3.34-3.44) in the AF cohort, 1.57 (95% CI, 1.39-1.74) in the AFL cohort, and 0.32 (95% CI, 0.29-0.35) in the control cohort, and they were significantly different from each other (Figure 2B). When we stratified the groups by CHA_2_DS_2_-VASc score, the IDs of hospitalization for heart failure increased with the level of CHA_2_DS_2_-VASc score in all 3 cohorts (Figure 4B). The IDs of hospitalization for heart failure in the AF and AFL cohorts were significantly higher across all levels of CHA_2_DS_2_-VASc compared with that in the control cohort (Figure 4B). The ID of hospitalization for heart failure was significantly higher in the AF cohort than in the AFL cohort across nearly all levels except at a CHA_2_DS_2_-VASc score of 0 (Figure 4B). In addition, the differences between the AFL and AF cohorts and between the AFL and control cohorts persisted as the CHA_2_DS_2_-VASc score increased.

### All-Cause Mortality

The IDs for all-cause mortality were 17.8 (95% CI, 17.7-17.9) in the AF cohort, 13.9 (95% CI, 13.4-14.4) in the AFL cohort, and 4.2 (95% CI, 4.1-4.4) in the control cohort, and there were significant differences among them (Figure 2C). When we stratified the groups by CHA_2_DS_2_-VASc score, the IDs of all-cause mortality increased with the level of CHA_2_DS_2_-VASc score in all 3 cohorts (Figure 4C). The IDs of all-cause mortality in the AF and AFL cohorts were significantly higher across all levels of CHA_2_DS_2_-VASc score compared with those in the control cohort (Figure 4C). The ID of all-cause mortality was significantly higher in the AF cohort than in the AFL cohort at CHA_2_DS_2_-VASc scores of 1, 2, and 3. In addition, the difference in ID between the AFL and control cohorts persisted, whereas the difference in the incidence of all-cause mortality between the AF and AFL cohorts decreased as the CHA_2_DS_2_-VASc score increased.

## Discussion

This nationwide cohort study found that patients with AF and AFL had significantly higher prevalence rates of comorbidities compared with matched control individuals. In addition, the ID of ischemic stroke in the AF cohort was significantly higher across all levels of CHA_2_DS_2_-VASc score compared with that of the matched controls, whereas the ID of ischemic stroke in the AFL cohort was only significantly higher at CHA_2_DS_2_-VASc scores of 5 to 9 compared with that in the control cohort. Moreover, the IDs of hospitalization for heart failure and all-cause mortality in the AF and AFL cohorts were significantly higher across all levels of CHA_2_DS_2_-VASc score compared with those in the control cohort.

### Real-World Clinical Implications of the AF, AFL, and Control Cohorts

In this study, the AF and AFL cohorts had significantly higher prevalence rates of comorbidities than did the control cohort, and the AF cohort had significantly higher prevalence rates of several comorbidities than did the AFL cohort. These differences in comorbidities among the 3 groups may have contributed to the different degrees of atrial myopathy, endocardial remodeling, and neurohumoral activation in the 3 cohorts and, thus, the different clinical outcomes. The risk of ischemic stroke among the patients with AFL was closer to that among the matched controls compared with the risk among the patients with AF (Figure 2A); this finding is consistent with that in the study by Biblo et al.^11^ In addition, the incidence of all-cause mortality in the AFL cohort was closer to that in the AF cohort than that in the matched control cohort (Figure 2C), whereas the incidence of hospitalization for heart failure in the AFL cohort was between that of the control and AF cohorts (Figure 2B). These findings in heart failure and all-cause mortality among the AF, AFL, and control cohorts were also reported in the Framingham Heart Study.^3^

### CHA_2_DS_2_-VASc Score on Clinical Outcomes

The CHA_2_DS_2_-VASc scoring system is used to predict the annual incidence of ischemic stroke,^12^ mortality, and heart failure^14,35^ in patients with AF and AFL. Therefore, we used the CHA_2_DS_2_-VASc scoring system to evaluate the incidences of ischemic stroke, hospitalization for heart failure, and all-cause mortality in the AF and AFL cohorts. Consistent with previous studies,^35,36^ the incidence rates of ischemic stroke, hospitalization for heart failure, and all-cause mortality increased with increasing CHA_2_DS_2_-VASc score in the AF and AFL cohorts. The incidence of ischemic stroke in the AFL cohort was higher than that in the control cohort but closer to that in the AF cohort with a high CHA_2_DS_2_-VASc score. The difference in hospitalization for heart failure among the 3 cohorts persisted as the CHA_2_DS_2_-VASc score increased. In terms of all-cause mortality, the difference between the AFL and control cohorts persisted across all levels of CHA_2_DS_2_-VASc score, whereas the difference between the AF and AFL cohorts decreased as the CHA_2_DS_2_-VASc score increased. Therefore, the CHA_2_DS_2_-VASc score had different implications for the clinical outcomes among the 3 cohorts. However, the CHA_2_DS_2_-VASc scoring system reflects underlying comorbidities, and more comorbidities are associated with higher mortality.^37^ Therefore, the difference in ischemic stroke, hospitalization for heart failure, and all-cause mortality between the AF and AFL cohorts may become less prominent as the CHA_2_DS_2_-VASc score increases (Figure 4).

### Clinical Implications in Preventing Ischemic Stroke

According to the current guidelines^7^ and a meta-analysis,^6^ patients with AFL should be treated in the same manner as patients with AF for preventing ischemic stroke. However, the observational studies in the meta-analysis were heterogeneous in their data and findings, and the largest study^11^ reported that patients with AFL had a higher risk of stroke compared with the controls but a lower risk compared with the patients with AF, which is similar to our findings. Therefore, our results suggest that the efficacy and safety of oral anticoagulants to reduce ischemic stroke in patients with AFL with the currently recommended CHA_2_DS_2_-VASc score should be reevaluated. According to the 2016 European Society of Cardiology guidelines for preventing ischemic stroke in patients with AF and AFL, anticoagulation therapy should be prescribed for patients with a CHA_2_DS_2_-VASc score of 2 or higher, and non–vitamin K oral anticoagulants should be considered for patients with a CHA_2_DS_2_-VASc score of 1 or higher because of net clinical benefits.^7,38,39^ In the current study, the ID of ischemic stroke at a CHA_2_DS_2_-VASc score of 1 in the AF cohort was similar to that at a CHA_2_DS_2_-VASc score of 2 in the AFL cohort, and the ID of ischemic stroke at a CHA_2_DS_2_-VASc score of 2 in the AF cohort was similar to that at a CHA_2_DS_2_-VASc score of 4 in the AFL cohort (Figure 3 and Figure 4A). In addition, 2 studies^34,39^ recommended prescribing anticoagulation therapy to patients with AF with a 1.7% or greater annual incidence of ischemic stroke. On the basis of several clinical trials, one of these studies^34^ recommended that non–vitamin K oral anticoagulants should be considered when the annual incidence of stroke is 0.9% or greater. Similarly, our results indicate that patients with AFL may be prescribed anticoagulants when the CHA_2_DS_2_-VASc score is 4 or higher (with an ID of 2.3%) and non–vitamin K oral anticoagulants when the CHA_2_DS_2_-VASc score is 2 or higher (with an ID of 1.0%) (Figure 3). However, from a statistical significance point of view, the incidences of ischemic stroke in the AF cohort across all levels of CHA_2_DS_2_-VASc and in the AFL cohort at a CHA_2_DS_2_-VASc score of 5 to 9 were significantly higher than in the control cohort. Thus, oral anticoagulants should be considered for patients with AFL and patients with AF when the CHA_2_DS_2_-VASc score is 5 or higher (Figure 4A).

### Limitations

There are several limitations to this retrospective cohort database study. First, AF was not subclassified into paroxysmal AF, persistent AF, and chronic AF, and AFL was not subclassified into typical and atypical AFL in the NHIRD. Although different types of AF and AFL may have different clinical outcomes, the clinical outcomes of different types of AF or AFL according to the CHA_2_DS_2_-VASc score have not been reported.

Second, although the accuracy of the diagnoses of AF and AFL and clinical outcomes based on an insurance database may not be the same as those from reviewing clinical records and relevant examination data, our prior study^30^ and others^29,32^ found a high positive predictive value and accuracy of insurance databases, and the large size of the database in this study should be sufficient to reach an accurate statistical conclusion.^31,40^

Third, selection bias may be possible in selecting patients at relatively low risk of ischemic stroke in our study because of the exclusion of any anticoagulation therapy during the observation period. However, in subgroup analysis for the excluded patients who received anticoagulation therapy during the observation period (n = 46 000), the annual ID of ischemic stroke was higher in the AF cohort (ID, 6.16; 95% CI, 6.04-6.29) than in the AFL cohort (ID, 2.65; 95% CI, 2.00-3.31) (eTable 5 in the Supplement), and no significant differences were found in baseline characteristics between the patients with AF and the patients with AFL who were prescribed anticoagulants after an ischemic stroke event after the index date (eTable 6 in the Supplement). Therefore, the exclusion of patients receiving any anticoagulation therapy during our observation period should have little effect on our results. In addition, although some evidence indicated no significant differences between rate control and rhythm control (focus of antiarrhythmic drugs) in patients with AF and AFL,^41,42^ we could not completely conclude that any antiarrhythmic drug had no influence on the study outcomes. Therefore, we performed a sensitivity analysis after excluding those taking an antiarrhythmic medication (eTable 7 in the Supplement), and the results indicate that the patterns of ischemic stroke, heart failure hospitalization, and all-cause mortality among the AFL, AFL, and matched control cohorts were the same as in our main analysis. Catheter ablation yields a high success rate for sinus conversion in patients with AFL,^43^ whereas it does not have a comparable success rate in patients with AF.^44^ Although rare evidence supports a correlation between catheter ablation and clinical outcomes in patients with AFL, we excluded such patients in our studies to minimize the possible bias. Electrical cardioversion is another issue for sinus conversion; however, a meta-analysis^45^ concluded that electrical cardioversion did not influence the incidence of stroke. In addition, the clinical presentation during electrical cardioversion and successful rate of electrical cardioversion could not be assessed, and we cannot clearly define whether the electrical cardioversion was performed for AFL or AF in the database study. Therefore, we did not include electrical cardioversion in our study.

Fourth, our study did not examine whether anticoagulation should not be used in patients with AFL and a CHA_2_DS_2_-VASc score of 4 or greater to prevent ischemic stroke. No data were available regarding the difference in ischemic stroke between patients with AFL with and without anticoagulation therapy at a CHA_2_DS_2_-VASc score of 4 or less, and additional studies are warranted to clarify this point. Fifth, although we could not entirely exclude AF events in the AFL cohort based on *ICD-9-CM* codes, we still found significant differences in the IDs of ischemic stroke, hospitalization for heart failure, and all-cause mortality among the 3 cohorts. Sixth, our study enrolled only Taiwanese participants, and we do not know whether our result could be extrapolated to non-Asian populations; therefore, further study should be conducted. Seventh, nonrandomized observational studies are prone to residual confounding or unmeasured confounding; however, our falsification analysis revealed no strong evidence of residual confounding in the comparison between the AF and AFL groups (eTable 8 in the Supplement).

## Conclusions

This large nationwide cohort study demonstrated different clinical outcomes in patients with AFL and AF compared with those without AF and AFL. The IDs of ischemic stroke in the AF cohort were significantly higher across all levels of the CHA_2_DS_2_-VASc score compared with the matched control cohort, whereas the IDs of ischemic stroke in the AFL cohort were only significantly higher at CHA_2_DS_2_-VASc scores of 5 to 9 compared with that in the control cohort. Moreover, the IDs of hospitalization for heart failure and all-cause mortality in the AF and AFL cohorts were significantly higher across all levels of the CHA_2_DS_2_-VASc score compared with those of the controls. Our study suggests that further research should be done to reevaluate the net clinical benefit of oral anticoagulants to prevent ischemic stroke in patients with AFL according to the currently recommended level of the CHA_2_DS_2_-VASc score.

## Notes

Supplement. **eTable 1.** Diagnoses Used to Define the Cohorts, Comorbidities, and Outcomes **eTable 2.** Anatomical Therapeutic Chemical (ATC) Codes of the Study Medications **eTable 3.** Medications Among the Atrial Fibrillation, Atrial Flutter and Matched Control Groups **eTable 4.** Detailed Information of Ischemic Stroke in the AF, AFL and Control Groups Stratified By CHA2DS2-VASc **eTable 5.** Baseline Characteristics and Ischemic Stroke Event of Population with Anticoagulation Therapy After Index Date **eTable 6.** Baseline Characteristics of Patients Who Were Prescribed Anticoagulants After Event of Ischemic Stroke After Index Date **eTable 7.** Sensitivity Analysis for Those Who Did Not Receive Any Antiarrhythmic Drugs After the Index Date **eTable 8.** Analysis of Falsification End-Point Using Hepatocellular Carcinoma as Outcome
