# Supplementary material for: Comparison of Clinical Outcomes Among Patients With Atrial Fibrillation or Atrial Flutter Stratified by CHA2DS2-VASc Score
Source: JAMA Netw Open. 2018 Aug 3;1(4):e180941. doi: 10.1001/jamanetworkopen.2018.0941 (PMC6324304; doi:10.1001/jamanetworkopen.2018.0941)
Supplement: Supplement. — eTable 1. Diagnoses Used to Define the Cohorts, Comorbidities, and Outcomes eTable 2. Anatomical Therapeutic Chemical (ATC) Codes of the Study Medications eTable 3. Medications Among the Atrial Fibrillation, Atrial Flutter and Matched Control Groups eTable 4. Detailed Information of Ischemic Stroke in the AF, AFL and Control Groups Stratified By CHA2DS2-VASc eTable 5. Baseline Characteristics and Ischemic Stroke Event of Population with Anticoagulation Therapy After Index Date eTable 6. Baseline Characteristics of Patients Who Were Prescribed Anticoagulants After Event of Ischemic Stroke After Index Date eTable 7. Sensitivity Analysis for Those Who Did Not Receive Any Antiarrhythmic Drugs After the Index Date eTable 8. Analysis of Falsification End-Point Using Hepatocellular Carcinoma as Outcome [file jamanetwopen-1-e180941-s001.pdf]

# Supplementary Online Content

Lin Y-S, Chen Y-L, Chen T-H, et al. Comparison of Clinical Outcomes Among Patients With Atrial Fibrillation or Atrial Flutter Stratified by $CHA_2DS_2$-VASc Score. *JAMA Netw Open.* 2018;1(4):e180941. doi:10.1001/jamanetworkopen.2018.0941

Supplement. eTable 1. Diagnoses Used to Define the Cohorts, Comorbidities, and Outcomes

eTable 2. Anatomical Therapeutic Chemical (ATC) Codes of the Study Medications

eTable 3. Medications Among the Atrial Fibrillation, Atrial Flutter and Matched Control Groups

eTable 4. Detailed Information of Ischemic Stroke in the AF, AFL and Control Groups Stratified By CHA2DS2-VASc

eTable 5. Baseline Characteristics and Ischemic Stroke Event of Population with Anticoagulation Therapy After Index Date

eTable 6. Baseline Characteristics of Patients Who Were Prescribed Anticoagulants After Event of Ischemic Stroke After Index Date

eTable 7. Sensitivity Analysis for Those Who Did Not Receive Any Antiarrhythmic Drugs After the Index Date

eTable 8. Analysis of Falsification End-Point Using Hepatocellular Carcinoma as Outcome

This supplementary material has been provided by the authors to give readers additional information about their work.

© 2018 Lin Y-S et al. *JAMA Network Open.*

**eTable 1. Diagnoses Used to Define the Cohorts, Comorbidities, and Outcomes**

| Disease | Criteria of Diagnosis | |
|---|---|---|
| **Study Cohorts** | | |
| Atrial fibrillation | Defined from diagnosis | ICD9-CM: 42731 |
| Atrial flutter | Defined from diagnosis | ICD9-CM: 42732 |
| **Excluding Diseases** | | |
| Hyperthyroidism | Defined from diagnosis plus treatment | ICD9-CM: 242 and any anti-thyroid drugs |
| Rheumatic heart disease | Defined from diagnosis | ICD9-CM:<br>394.0, 394.1, 394.2, 395, 398.9 |
| Sepsis | Defined from diagnosis during hospitalization | ICD9-CM: 038, 790.7 |
| **Comorbidities** | | |
| Hypertension | Defined from diagnosis plus treatment | 401, 402, 403, 404, 405<br>and any anti-hypertension drugs |
| Diabetes mellitus | Defined from diagnosis plus treatment | 250<br>and any oral hypoglycemic drugs and insulin |
| Ischemic heart disease | Defined from diagnosis | 410 ~414 |
| Heart failure | Defined from diagnosis | 428 |
| Dyslipidemia | Defined from diagnosis plus treatment | 272 and any lipid-lowing agents |
| Gout | Defined from diagnosis | 274 |
| Chronic obstructive pulmonary disease | Defined from diagnosis | 491, 492, 496 |
| Peripheral arterial disease | Defined from diagnosis | 440.0, 440.2, 440.3, 440.8, 440.9, 443,<br>444.0, 444.22, 444.8, 447.8 and 447.9 |
| Chronic kidney disease | Defined from diagnosis | 580 ~589, 403 ~404<br>016.0, 095.4, 236.9 250.4, 274.1, 442.1, 447.3,<br>440.1, 572.4, 642.1, 646.2 753.1 |

© 2018 Lin Y-S et al. *JAMA Network Open.*

| | | |
|---|---|---|
| | | 283.11, 403.01,and 404.02, 446.21 |
| Dialysis | Defined from diagnosis in catastrophic registry data | 585 |
| Abnormal liver function | Defined from diagnosis | 070 , 456.0–456.2, 570, 571,<br>572.2–572.8, 573, V42.7 |
| Malignancy | Defined from diagnosis in catastrophic registry data | 140 ~208 |
| **History of Events** | | |
| Systemic thromboembolism | Defined from diagnosis in hospitalization | 444.22, 444.81, 444.21,<br>557.0, 557.9, 557.1<br>362.30, 362.34, 593.81, 444.89, 433.8,<br>444.9 |
| Myocardial infarction | Defined from diagnosis in hospitalization | 410 , 412 |
| Ischemic stroke | Defined from diagnosis in hospitalization | 433 ~437 |
| **Outcomes** | | |
| Ischemic stroke | Defined from principle diagnosis of hospitalization | 433 ~437 |
| Heart failure | Defined from principle diagnosis of hospitalization | 428 |

© 2018 Lin Y-S et al. *JAMA Network Open.*

**eTable 2. Anatomical Therapeutic Chemical (ATC) Codes of the Study Medications**

| Medication[a] | ATC code | Medication[a] | ATC Code |
|---|---|---|---|
| Angiotensin converting enzyme inhibitors/angiotensin II receptor blockers | C09 | Dipeptidyl peptidase 4 inhibitors | A10BH |
| ß-blockers | C07 | Biguanides | A10BA |
| Calcium channel blockers | C08 | Glinides | A10BX02, A10BX03 |
| Diuretics | C03 | Alpha-glucosidase inhibitors | A10BF |
| Statins | C10AA | Sulfonylurea | A10BB |
| Antiplatelet agents | B01AC | Thiazolidinedione | A10BG02, A10BG03 |
| Warfarin | B01AA | Insulin | A10A |
| Digoxin | C01AA05 | | |

[a]The usage of these medications was defined as being prescribed with the medication for at least 3 months.

© 2018 Lin Y-S et al. *JAMA Network Open.*

**eTable 3. Medications Among the Atrial Fibrillation, Atrial Flutter and Matched Control Groups**

| **Medications (No. (%))** | **Matched control (*n* = 24,484)** | **AFL (*n* = 6,121)** | **AF (*n* = 188,811)** |
|---|---|---|---|
| Angiotensin converting enzyme inhibitors / angiotensin II receptor blockers | 4,568 (18.7) | 1,867 (30.5)[a] | 68,671 (36.4)[a b] |
| Calcium channel blockers | 4,888 (20.0) | 1,344 (22.0)[a] | 46,407 (24.6)[a b] |
| ß-blockers | 3,560 (14.5) | 1,959 (32.0)[a] | 54,890 (29.1)[a b] |
| Digoxin | 221 ( .9) | 624 (10.2)[a] | 33,498 (17.7)[a b] |
| Diuretics | 972 (4.0) | 995 (16.3)[a] | 39,530 (20.9)[a b] |
| Spironolactone | 180 (0.7) | 269 (4.4)[a] | 10,519 (5.6)[a b] |
| Dipeptidyl peptidase 4 inhibitors | 162 (0.7) | 86 (1.4)[a] | 2,523 (1.3)[a] |
| Statins | 1,729 (7.1) | 631 (10.3)[a] | 18,172 (9.6)[a] |
| Biguanides | 2,053 (8.4) | 586 (9.6)[a] | 17,457 (9.2)[a] |
| Sulfonylurea | 2,279 (9.3) | 649 (10.6)[a] | 19,518 (10.3)[a] |
| Thiazolidinedione | 382 (1.6) | 101 (1.7) | 2,595 (1.4) |
| Insulin | 333 (1.4) | 225 (3.7)[a] | 6,509 (3.4)[a] |
| Anti-arrhythmic drugs | | | |
| Amiodarone/Dronedarone | 73 (0.3) | 815 (13.3)[a] | 28,497 (15.1)[a b] |
| Propafenone | 0 (0.0) | 287 (4.7)[a] | 9,385 (5.0)[a] |
| Sotalol | 0 (0.0) | 13 (0.2)[a] | 199 (0.1)[a b] |
| Flecainide | 0 (0.0) | 9 (0.15)[a] | 116 (0.06)[a b] |
| Antiplatelet | 3,449 (14.1) | 1,940 (31.7)[a] | 80,518 (42.6)[a b] |

Data are presented as mean ± SD or number (percentage);

Abbreviations: AF: atrial fibrillation; AFL: atrial flutter;

The character "[a]" and " [b]" denotes a significant *post hoc* comparison versus control and AFL respectively.

© 2018 Lin Y-S et al. *JAMA Network Open.*

## eTable 4. Detailed Information Of Ischemic Stroke In The AF, AFL And Control Groups Stratified By CHA2DS2-VASc

| | Incidence density (95% CI) | | | AF vs. AFL | | AFL vs. Control | | AF vs. Control | |
|---|---|---|---|---|---|---|---|---|---|
| CHA2DS2-VASc | Matched controls (*n* = 24,484) | AFL (*n* = 6,121) | AF (*n* = 188,811) | HR (95% CI) | *P* value | HR (95% CI) | *P* value | HR (95% CI) | *P* value |
| 0 | 0.19 (0.13–0.25) | 0.39 (0.13–0.64) | 0.65 (0.57–0.72) | 1.64 (0.85, 3.19) | .14 | 1.98 (0.96, 4.07) | .064 | 3.25 (2.34, 4.51) | <.001 |
| 1 | 0.44 (0.36–0.52) | 0.40 (0.21–0.59) | 1.14 (1.06–1.22) | 2.84 (1.76, 4.60) | <.001 | 0.87 (0.53, 1.45) | .60 | 2.49 (2.06, 3.00) | <.001 |
| 2 | 1.02 (0.90–1.14) | 1.02 (0.69–1.34) | 2.30 (2.20–2.40) | 2.22 (1.60, 3.07) | <.001 | 0.94 (0.67, 1.33) | .73 | 2.08 (1.83, 2.37) | <.001 |
| 3 | 1.23 (1.07–1.38) | 1.37 (0.97–1.77) | 3.17 (3.06–3.28) | 2.26 (1.69, 3.02) | <.001 | 1.05 (0.77, 1.44) | .76 | 2.37 (2.09, 2.70) | <.001 |
| 4 | 1.78 (1.54–2.02) | 2.30 (1.72–2.88) | 3.82 (3.69–3.95) | 1.63 (1.27, 2.10) | <.001 | 1.20 (0.90, 1.60) | .21 | 1.96 (1.71, 2.25) | <.001 |
| 5-9 | 2.83 (2.48–3.18) | 3.99 (3.22–4.76) | 5.38 (5.24–5.52) | 1.34 (1.10, 1.62) | .004 | 1.29 (1.02, 1.62) | .033 | 1.72 (1.51, 1.95) | <.001 |

Abbreviations: AF: atrial fibrillation; AFL: atrial flutter; HR: hazard ratio; CI: confidence interval.

© 2018 Lin Y-S et al. *JAMA Network Open.*

## eTable 5. Baseline Characteristics and Ischemic Stroke Event of Population with Anticoagulation Therapy After Index Date

| **Variables** | **Total (*n* = 46,000)** | **AFL (*n* = 703)** | **AF (*n* = 45,297)** |
|---|---|---|---|
| Age (years; mean ± SD) * | 70.5±11.9 | 67.0±13.8 | 70.5±11.8 |
| Age group (No. (%))* | | | |
| < 65 years | 13,275 (28.9) | 279 (39.7) | 12,996 (28.7) |
| 65~74 years | 14,141 (30.7) | 202 (28.7) | 13,939 (30.8) |
| ≥ 75 years | 18,584 (40.4) | 222 (31.6) | 18,362 (40.5) |
| Gender (No. (%))* | | | |
| Male | 26,124 (56.8) | 466 (66.3) | 25,658 (56.6) |
| Female | 19,876 (43.2) | 237 (33.7) | 19,639 (43.4) |
| Comorbidities (No. (%)) | | | |
| Hypertension | 29,008 (63.1) | 426 (60.6) | 28,582 (63.1) |
| Ischemic heart disease | 18,045 (39.2) | 294 (41.8) | 17,751 (39.2) |
| Diabetes mellitus* | 8,655 (18.8) | 168 (23.9) | 8,487 (18.7) |
| Chronic obstructive pulmonary disease | 6,756 (14.7) | 92 (13.1) | 6,664 (14.7) |
| Dyslipidemia* | 6,233 (13.6) | 114 (16.2) | 6,119 (13.5) |
| Heart failure* | 5,561 (12.1) | 103 (14.7) | 5,458 (12.0) |
| Gout | 5,549 (12.1) | 91 (12.9) | 5,458 (12.0) |
| Peripheral arterial disease | 1,363 (3.0) | 25 (3.6) | 1,338 (3.0) |
| Renal status* | | | |
| Non-chronic kidney disease | 40,986 (89.1) | 584 (83.1) | 40,402 (89.2) |
| Chronic kidney disease without dialysis | 4,198 (9.1) | 90 (12.8) | 4,108 (9.1) |
| Chronic kidney disease with dialysis | 816 (1.8) | 29 (4.1) | 787 (1.7) |
| Immune disease* | 719 (1.6) | 18 (2.6) | 701 (1.5) |

© 2018 Lin Y-S et al. *JAMA Network Open.*

| | | | |
|---|---|---|---|
| Abnormal liver function | 4,578 (10.0) | 72 (10.2) | 4,506 (9.9) |
| Malignancy | 2,411 (5.2) | 41 (5.8) | 2,370 (5.2) |
| History of disease (No. (%)) | | | |
| Prior stroke or systemic thromboembolism | 8,137 (17.7) | 126 (17.9) | 8,011 (17.7) |
| Prior stroke | 6,736 (14.6) | 90 (12.8) | 6,646 (14.7) |
| Old myocardial infarction* | 1,871 (4.1) | 57 (8.1) | 1,814 (4.0) |
| $CHA_2DS_2$-VASc score ( mean ± SD) * | 3.3±1.8 | 3.0±1.9 | 3.3±1.8 |
| $CHA_2DS_2$-VASc group* | | | |
| 0 | 2,889 (6.3) | 53 (7.5) | 2,836 (6.3) |
| 1 | 5,563 (12.1) | 114 (16.2) | 5,449 (12.0) |
| 2 | 7,836 (17.0) | 129 (18.3) | 7,707 (17.0) |
| 3 | 9,702 (21.1) | 135 (19.2) | 9,567 (21.1) |
| 4 | 8,805 (19.1) | 116 (16.5) | 8,689 (19.2) |
| 5~9 | 11,205(24.4) | 156(22.2) | 11,049(24.4) |
| **Events of ischemic stroke after index date** | | | |
| Event rate | 9,645 (20.97) | 63 (8.96) | 9,582 (21.15) |
| Incidence density | 6.1 (6.0–6.2) | 2.7 (2.0–3.3) | 6.2 (6.0–6.3) |

Data are presented as mean ± SD or number (percentage);

*indicates $P < 0.05$ between the two groups;

Abbreviations: AF: atrial fibrillation; AFL: atrial flutter.

© 2018 Lin Y-S et al. *JAMA Network Open.*

**eTable 6. Baseline Characteristics of Patients Who Were Prescribed Anticoagulants After Event of Ischemic Stroke After Index Date**

| **Variables** | **Total (*n* = 5,080)** | **AFL (*n* = 33)** | **AF (*n* = 5,047)** |
|---|---|---|---|
| Age (years; mean ± SD) | 71.1±10.3 | 67.6±12.3 | 71.1±10.3 |
| Age group (No. (%)) | | | |
| < 65 years | 1,272 (25.0) | 13 (39.4) | 1,259 (24.9) |
| 65~74 years | 1,791 (35.3) | 10 (30.3) | 1,781 (35.3) |
| ≥ 75 years | 2,017 (39.7) | 10 (30.3) | 2,007 (39.8) |
| Gender (No. (%)) | | | |
| Male | 2,696 (53.1) | 23 (69.7) | 2,673 (53.0) |
| Female | 2,384 (46.9) | 10 (30.3) | 2,374 (47.0) |
| Comorbidities (No. (%)) | | | |
| Hypertension | 3,237 (63.7) | 24 (72.7) | 3,213 (63.7) |
| Ischemic heart disease | 2,057 (40.5) | 14 (42.4) | 2,043 (40.5) |
| Diabetes mellitus | 987 (19.4) | 10 (30.3) | 977 (19.4) |
| Dyslipidemia | 679 (13.4) | 5 (15.2) | 674 (13.4) |
| Gout* | 623 (12.3) | 8 (24.2) | 615 (12.2) |
| Chronic obstructive pulmonary disease | 659 (13.0) | 3 (9.1) | 656 (13.0) |
| Heart failure | 434 (8.5) | 4 (12.1) | 430 (8.5) |
| Peripheral arterial disease | 128 (2.5) | 2 (6.1) | 126 (2.5) |
| Renal status | | | |
| Non-chronic kidney disease | 4,616 (90.9) | 28 (84.8) | 4,588 (90.9) |
| Chronic kidney disease without dialysis | 412 (8.1) | 4 (12.1) | 408 (8.1) |
| Chronic kidney disease with dialysis | 52 (1.0) | 1 (3.0) | 51 (1.0) |

© 2018 Lin Y-S et al. *JAMA Network Open.*

| | | | |
|---|---|---|---|
| Immune disease | 66 (1.3) | 0 (0.0) | 66 (1.3) |
| Abnormal liver function | 444 (8.7) | 4 (12.1) | 440 (8.7) |
| Malignancy | 185 (3.6) | 1 (3.0) | 184 (3.6) |
| History of disease (No. (%)) | | | |
| Prior stroke or systemic thromboembolism | 823 (16.2) | 8 (24.2) | 815 (16.1) |
| Prior stroke | 765 (15.1) | 8 (24.2) | 757 (15.0) |
| Old myocardial infarction* | 176 (3.5) | 5 (15.2) | 171 (3.4) |
| $CHA_2DS_2$-VASc score ( mean ± SD) | 3.3±1.7 | 3.3±1.8 | 3.3±1.7 |
| $CHA_2DS_2$-VASc group | | | |
| 0 | 278 (5.5) | 2 (6.1) | 276 (5.5) |
| 1 | 539 (10.6) | 4 (12.1) | 535 (10.6) |
| 2 | 894 (17.6) | 4 (12.1) | 890 (17.6) |
| 3 | 1,126 (22.2) | 9 (27.3) | 1,117 (22.1) |
| 4 | 1,044 (20.6) | 5 (15.2) | 1,039 (20.6) |
| 5~9 | 1,199(23.6) | 9(23.3) | 1,190(23.6) |

Data are presented as mean ± SD or number (percentage);

*indicates $P < 0.05$ between the two groups;

Abbreviations: AF: atrial fibrillation; AFL: atrial flutter.

© 2018 Lin Y-S et al. *JAMA Network Open.*

**eTable 7. Sensitivity Analysis For Those Who Did Not Receive Any Antiarrhythmic Drugs After The Index Date**

| | Incidence density (95% CI) | | | AF vs. AFL | | AFL vs. Matched controls | | AF vs. Matched controls | |
|---|---|---|---|---|---|---|---|---|---|
| Outcome / CHA2DS2-VASc | Matched controls (*n* = 24,411) | AFL (*n* = 5,021) | AF (*n* = 151,640) | HR (95% CI) | *P* value | HR (95% CI) | *P* value | HR (95% CI) | *P* value |
| Ischemic stroke | | | | | | | | | |
| 0 | 0.19 (0.13–0.25) | 0.47 (0.16–0.77) | 0.66 (0.58–0.75) | 1.40 (0.72, 2.73) | .32 | 2.37 (1.15, 4.88) | .019 | 3.33 (2.39, 4.64) | <.001 |
| 1 | 0.44 (0.36–0.52) | 0.41 (0.20–0.61) | 1.19 (1.09–1.28) | 2.88 (1.73, 4.80) | <.001 | 0.90 (0.53, 1.54) | .70 | 2.59 (2.14, 3.13) | <.001 |
| 2 | 1.02 (0.90–1.15) | 1.11 (0.73–1.50) | 2.52 (2.40–2.64) | 2.21 (1.56, 3.14) | <.001 | 1.02 (0.71, 1.48) | .90 | 2.26 (1.99, 2.58) | <.001 |
| 3 | 1.23 (1.08–1.38) | 1.30 (0.86–1.74) | 3.42 (3.29–3.54) | 2.55 (1.81, 3.57) | <.001 | 1.002 (0.70, 1.43) | .99 | 2.55 (2.24, 2.90) | <.001 |
| 4 | 1.78 (1.54–2.02) | 2.29 (1.65–2.93) | 4.04 (3.89–4.19) | 1.72 (1.30, 2.29) | <.001 | 1.20 (0.88, 1.63) | .26 | 2.06 (1.79, 2.37) | <.001 |
| 5-9 | 2.85 (2.49–3.20) | 4.17 (3.30–5.04) | 5.66 (5.50–5.83) | 1.33 (1.08, 1.65) | .008 | 1.34 (1.05, 1.71) | .019 | 1.78 (1.57, 2.02) | <.001 |
| Hospitalization for heart failure | | | | | | | | | |
| 0 | 0.02 (0.00–0.04) | 0.47 (0.16–0.77) | 0.59 (0.51–0.67) | 1.22 (0.62, 2.37) | .56 | 19.39 (6.50, 57.85) | <.001 | 23.60 (9.72, 57.31) | <.001 |
| 1 | 0.07 (0.04–0.10) | 0.19 (0.05–0.33) | 0.95 (0.87–1.03) | 4.94 (2.34, 10.41) | <.001 | 2.48 (1.06, 5.84) | .037 | 12.25 (7.92, 18.95) | <.001 |

© 2018 Lin Y-S et al. *JAMA Network Open.*

| | | | | | | | | | | | | |
|---|---|---|---|---|---|---|---|---|---|---|---|---|
| 2 | 0.20 (0.15–0.26) | 1.01 (0.64–1.38) | 1.90 (1.79–2.00) | | 1.82 (1.26, 2.63) | .001 | | 4.59 (2.92, 7.23) | <.001 | | 8.36 (6.34, 11.04) | <.001 |
| 3 | 0.41 (0.32–0.50) | 1.19 (0.77–1.61) | 3.23 (3.11–3.36) | | 2.61 (1.83, 3.71) | <.001 | | 2.65 (1.76, 3.99) | <.001 | | 6.90 (5.57, 8.54) | <.001 |
| 4 | 0.71 (0.57–0.86) | 2.21 (1.58–2.83) | 4.38 (4.23–4.54) | | 1.92 (1.45, 2.56) | <.001 | | 2.82 (1.98, 4.01) | <.001 | | 5.42 (4.38, 6.70) | <.001 |
| 5-9 | 1.33 (1.09–1.56) | 4.75 (3.82–5.67) | 6.95 (6.77–7.13) | | 1.42 (1.17, 1.72) | <.001 | | 3.11 (2.39, 4.05) | <.001 | | 4.41 (3.69, 5.28) | <.001 |
| All-cause mortality | | | | | | | | | | | | |
| 0 | 0.63 (0.53–0.74) | 4.75 (3.79–5.72) | 4.82 (4.59–5.04) | | 0.98 (0.79, 1.21) | .84 | | 7.21 (5.54, 9.38) | <.001 | | 7.06 (5.93, 8.40) | <.001 |
| 1 | 1.40 (1.27–1.54) | | 6.72 (6.51–6.93) | | 1.43 (1.23, 1.66) | <.001 | | 3.13 (2.62, 3.74) | <.001 | | 4.47 (4.04, 4.95) | <.001 |
| 2 | 4.94 (4.67–5.21) | 12.44 (11.16–13.72) | 15.85 (15.57–16.14) | | 1.25 (1.12, 1.38) | <.001 | | 2.30 (2.05, 2.59) | <.001 | | 2.87 (2.71, 3.04) | <.001 |
| 3 | 6.15 (5.82–6.48) | 16.69 (15.15–18.24) | 20.70 (20.39–21.00) | | 1.20 (1.09, 1.32) | <.001 | | 2.47 (2.22, 2.76) | <.001 | | 2.97 (2.81, 3.14) | <.001 |
| 4 | 7.38 (6.90–7.86) | 21.87 (19.94–23.81) | 23.52 (23.17–23.87) | | 1.05 (0.96, 1.15) | .26 | | 2.71 (2.42, 3.02) | <.001 | | 2.85 (2.66, 3.04) | <.001 |
| 5-9 | 10.89 (10.22–11.55) | 31.78 (29.46–34.09) | 32.91 (32.53–33.28) | | 1.02 (0.95, 1.10) | .62 | | 2.61 (2.37, 2.87) | <.001 | | 2.65 (2.49, 2.82) | <.001 |

Abbreviations: AF: atrial fibrillation; AFL: atrial flutter; HR: hazard ratio; CI: confidence interval.

© 2018 Lin Y-S et al. *JAMA Network Open.*

## eTable 8. Analysis Of Falsification End-Point Using Hepatocellular Carcinoma As Outcome

| | Incidence density (95% CI) | | AF vs. AFL | |
|---|---|---|---|---|
| CHA2DS2-VASc | AFL (*n* = 6,121) | AF (*n* = 188,811) | HR (95% CI) | *P* value |
| 0 | 0.04 (-0.04–0.13) | 0.23 (0.19–0.28) | 5.41 (0.75, 38.74) | .09 |
| 1 | 0.18 (0.06–0.31) | 0.30 (0.26–0.34) | 1.60 (0.79, 3.24) | .19 |
| 2 | 0.22 (0.07–0.37) | 0.36 (0.32–0.40) | 1.62 (0.81, 3.27) | .18 |
| 3 | 0.44 (0.22–0.66) | 0.37 (0.34–0.41) | 0.83 (0.50, 1.39) | .48 |
| 4 | 0.32 (0.11–0.53) | 0.40 (0.36–0.44) | 1.21 (0.63, 2.35) | .57 |
| 5-9 | 0.43 (0.19–0.68) | 0.32 (0.29–0.35) | 0.74 (0.42, 1.31) | .30 |

Abbreviations: AF: atrial fibrillation; AFL: atrial flutter; HR: hazard ratio; CI: confidence interval.

© 2018 Lin Y-S et al. *JAMA Network Open.*
